# Intra-Tumor Heterogeneity of Pancreatic Ductal Adenocarcinoma (PDAC)—Microenvironmental Interaction and Precision Immunotherapy Strategies: A Multi-Omics-Based Integrated Perspective

**DOI:** 10.3390/ijms27135682

**Published:** 2026-06-24

**Authors:** Boyeon Kim, Jee-Hyung Lee

**Affiliations:** 1 Division of Medical Oncology, Department of Internal Medicine, Korea University College of Medicine, Seoul 02841, Republic of Korea; 2 Cancer Institute, Korea University, Seoul 02841, Republic of Korea; 3 Center for Organoid Research & Development, Korea University Anam Hospital, Korea University College of Medicine, Seoul 02841, Republic of Korea; 4 Center for Innovative Drug Discovery, Korea University Anam Hospital, Korea University College of Medicine, Seoul 02841, Republic of Korea; 5 Advanced Medical Imaging Institute, Korea University College of Medicine, Seoul 02841, Republic of Korea

**Keywords:** pancreatic ductal adenocarcinoma, intratumoral heterogeneity, tumor microenvironment, immune exclusion, immunotherapy resistance, cancer-associated fibroblasts, spatial transcriptomics, patient-derived organoids, metabolic plasticity, precision immuno-oncology

## Abstract

Pancreatic ductal adenocarcinoma (PDAC) remains among the most therapeutically intractable malignancies, with a 5-year survival rate of approximately 10% and near-universal resistance to immune checkpoint inhibitor (ICI) therapy. This refractoriness arises from the convergence of pronounced intratumoral heterogeneity (ITH) and a profoundly immunosuppressive tumor microenvironment (TME), which together configure PDAC as a prototypical immune-excluded tumor. Beyond low tumor mutational burden, PDAC exhibits layered genetic, epigenetic, transcriptional, and metabolic heterogeneity that enables rapid adaptation and immune evasion under selective pressure, while dense desmoplastic stroma, cancer-associated fibroblasts (CAFs), and immunosuppressive immune populations collectively impose formidable physical and immunologic barriers to antitumor immunity. In this review, we synthesize multi-omics, spatial transcriptomic, and immunologic evidence to elucidate how ITH and the TME dynamically interact to reinforce immune resistance. We examine reciprocal crosstalk mechanisms—including immune-driven clonal selection, interclonal cooperation, metabolic niche specialization, and metabolic–epigenetic coupling—and discuss emerging platforms such as single-cell spatial omics, patient-derived organoid immune co-culture systems, and longitudinal circulating tumor DNA monitoring that enable high-resolution mapping of ITH–TME dynamics. Finally, we evaluate ITH–TME-guided combination therapeutic strategies targeting oncogenic drivers, stromal architecture, myeloid suppression, and metabolic checkpoints, and propose a prioritized framework for near-term and speculative clinical translation in PDAC.

## 1. Introduction

PDAC carries a 5-year survival rate of approximately 10%, reflecting early systemic dissemination and intrinsic resistance to multimodal therapy [1,2]. Although immune checkpoint inhibitors (ICIs) have demonstrated substantial benefits in several solid tumors such as melanoma (e.g., ORR ~58% with nivolumab plus ipilimumab; CheckMate 067) and non-small cell lung cancer with high PD-L1 expression (ORR ~45% with pembrolizumab monotherapy; KEYNOTE-024) [3,4], their therapeutic impact in PDAC remains markedly limited. For example, a phase II trial of ipilimumab monotherapy in advanced PDAC demonstrated virtually no objective responses, while dual checkpoint blockade with durvalumab plus tremelimumab also failed to produce meaningful survival improvement in unselected metastatic PDAC cohorts [5,6]. These disappointing clinical outcomes emphasize that checkpoint inhibition alone is insufficient to overcome the multilayered immune resistance mechanisms characteristic of PDAC [7]. Clinically meaningful responses are predominantly confined to a rare subset of PDAC cases exhibiting microsatellite instability–high (MSI-H) or mismatch repair-deficient (dMMR) phenotypes [8,9,10].

The therapeutic failure of PDAC is multifactorial and encompasses at least six major mechanistic axes: (1) severe tumor hypoxia resulting from vascular collapse within the desmoplastic stroma; (2) dense desmoplasia (constituting >50% of tumor volume), primarily driven by activated CAFs and hyaluronan-rich ECM that generates elevated interstitial fluid pressure and impedes drug penetration; (3) multidrug resistance protein (MDR) overexpression and drug efflux in tumor cells; (4) late diagnosis, with approximately 80–85% of PDAC patients presenting with locally advanced or metastatic disease at the time of diagnosis; (5) the absence of broadly effective KRAS-targeted inhibitors, as KRAS mutations (present in ~90% of PDAC) have only recently become partially addressable; and (6) profound ITH and an immunosuppressive TME, which together prevent effective immune-mediated tumor clearance [1,2,11,12,13,14,15,16,17]. Each of these barriers likely contributes independently and synergistically to treatment failure, and a comprehensive understanding of PDAC resistance must account for all of them. However, the present review specifically focuses on axes (5) and (6)—namely, the interplay between ITH and the TME—as these represent the mechanistic foundation upon which precision immunotherapy strategies must be built, and constitute an area of rapidly evolving translational research [11,12,13,18,19,20,21,22,23]. The desmoplastic stroma, driven by cancer-associated fibroblasts (CAFs) and excessive extracellular matrix (ECM) deposition, creates both physical and biophysical barriers that impede immune cell infiltration [14,15,16]. Concurrently, abundant immunosuppressive populations, including regulatory T-cells (Tregs), tumor-associated macrophages (TAMs), and myeloid-derived suppressor cells (MDSCs), reinforce immune evasion [24,25,26]. Spatial profiling studies have revealed that a substantial proportion (approximately 60–90%) of PDAC lesions exhibit immune-excluded or immune-desert architectures, with truly inflamed phenotypes being relatively uncommon [27,28,29].

In this review, we synthesize current multi-omics, spatial transcriptomic, and immunologic evidence to examine how ITH and TME co-evolve to mediate immune resistance in PDAC [18,19,20]. We integrate the genetic, epigenetic, metabolic, and stromal dimensions to outline the mechanistic basis of immune evasion and discuss emerging therapeutic strategies aimed at overcoming the intrinsic and microenvironment-driven barriers to effective immunotherapy [21,22,23].

## 2. Highly Immunosuppressive PDAC Tumor Microenvironment (TME)

The TME of PDAC represents the dominant structural barrier to immunotherapy, operating through three hierarchically organized mechanisms. First, CAF-driven desmoplasia generates a dense fibrotic ECM that physically excludes immune cells, constituting 50–80% of the tumor volume and elevating interstitial fluid pressure (IFP) to levels that compress the tumor vasculature [30,31,32,33,34]. Second, this physical exclusion is compounded by a multilayered immunosuppressive cell network comprising TAMs, MDSCs, and Tregs that actively suppress antitumor immunity via TGF-β, IL-10, and VEGF [24,25,26,35,36]. Third, spatial heterogeneity of these TME components within single tumors—giving rise to immune-desert, immune-excluded, and rare inflamed niches—produces mosaic immune architectures that resist uniform therapeutic intervention [20,28]. These three mechanisms are not parallel but hierarchical: stromal exclusion is primary, myeloid suppression is secondary, and spatial heterogeneity represents the emergent consequence of the first two. Understanding this hierarchy is clinically important: strategies that target myeloid populations without first disrupting the physical barrier are unlikely to achieve durable T-cell infiltration [22,23,37].

### 2.1. Extracellular Matrix (ECM) and the Desmoplastic Fibrosis Barrier

A defining hallmark of PDAC is its extensive desmoplastic reaction driven by activated CAFs and excessive ECM deposition [30,31]. This fibrotic network frequently constitutes the majority of the tumor volume and creates a rigid hypovascular architecture that limits immune cell access [32]. Dense collagen I, hyaluronic acid, and other ECM components increase the interstitial fluid pressure, compress the intratumoral vasculature, and generate hypoxic and metabolically stressed niches [33,34]. These stromal conditions activate mechanotransduction pathways, including FAK–Src and YAP/TAZ, which promote tumor cell survival programs and reinforce immune exclusion [38,39]. Hypoxia induces PD-L1 expression and metabolic adaptations that impair T-cell function [40,41,42]. Collectively, the desmoplastic ECM serves as a central physical and biochemical barrier that configures the immunosuppressive TME in PDAC [43].

#### 2.1.1. Composition of Dense Fibrous ECM (Desmoplasia) and Physical Restriction of T-Lymphocyte Infiltration

The TME of PDAC is distinguished by a prominent desmoplastic reaction comprising dense ECM deposition and the accumulation of CAFs [30,31,32]. This robust fibrotic stroma constitutes 50–80% of the total tumor volume, with the degree of fibrosis serving as an independent prognostic factor that negatively correlates with patient survival [33,44].

The ECM is primarily composed of collagen (particularly type I collagen), and non-collagen components such as hyaluronic acid. Excessive ECM deposition physically restricts the infiltration and proliferation of immune cells, including cytotoxic T lymphocytes (CTLs), and simultaneously acts as a major barrier to efficient intratumoral delivery of systemic anticancer drugs [33,34,44].

#### 2.1.2. High Interstitial Pressure, Hypoxia, and Acidity-Driven Mechanisms

Excessive ECM accumulation elevates interstitial fluid pressure, mechanically compressing intratumoral blood vessels, and reducing perfusion [33,34]. This results in hypoxia and acidification (low pH) of the tumor tissue [40]. The notion that hypoxia drives FOXP3 upregulation and Treg stabilization via HIF-1α is a concept extrapolated primarily from non-PDAC tumor systems; direct causal evidence for this pathway in PDAC is currently absent. Existing PDAC data demonstrate hypoxia-associated enrichment of immunosuppressive cell states, but the precise molecular pathway linking HIF-1α to FOXP3 induction in PDAC has not been defined experimentally. Existing PDAC studies have primarily shown hypoxia-associated enrichment of immunosuppressive cell states rather than a defined molecular pathway. Therefore, statements linking hypoxia to FOXP3 upregulation in PDAC should be interpreted as extrapolated concepts requiring experimental validation [45]. Concurrently, HIF-1α has been reported to increase PD-L1 expression in several tumor types, including PDAC in some studies; however, results vary across models [40,45]. Additionally, hypoxia induces HIF-1α–dependent metabolic reprogramming, which suppresses cytotoxic T-cell function through lactate accumulation and activation of the kynurenine pathway [41,42].

#### 2.1.3. Matrix Stiffness and FAK/YAP–TAZ-Mediated Mechanotransduction

ECM accumulation substantially increases the stiffness of the tumor matrix [38]. This increased stiffness triggers mechanotransduction via Yes-associated protein (YAP)/transcriptional coactivator with PDZ-binding motif (TAZ) and focal adhesion kinase (FAK)–Src signaling pathways, enhancing tumor-cell proliferation and resistance to apoptosis [38,39]. FAK, a non-receptor tyrosine kinase that transduces ECM-derived signals, is a key mediator of tumor–ECM interactions. In human PDAC, elevated FAK activity correlates with greater fibrosis and reduced CD8^+^ cytotoxic T-cell infiltration, thereby reinforcing an immunosuppressive microenvironment [39]. FAK activation also promotes fibroblast activation and ECM remodeling, which accelerates desmoplastic stromal expansion, contributing to both immune and therapeutic resistance [38,39].

### 2.2. Immunosuppressive Immune-Cell Landscape

The TME of PDAC contains numerous immunosuppressive cell populations that contribute to T-cell dysfunction and therapeutic resistance [35]. TAMs adopt predominantly M2-like, regulatory phenotypes and secrete IL-10, transforming growth factor-beta (TGF-β), and vascular endothelial growth factor (VEGF), thereby promoting immune tolerance and angiogenesis [36]. MDSCs are recruited by tumor- and CAF-derived factors, such as granulocyte-macrophage colony-stimulating factor (GM-CSF), IL-6, and CCL2, and profoundly suppress T-cell proliferation [46]. Tregs further amplify immunosuppression through the production of TGF-β and IL-10 [45]. Collectively, these cell populations constitute a multilayered immunoregulatory network that restricts effective antitumor immunity in PDAC.

#### 2.2.1. Accumulation and Roles of Regulatory T-Cells, Tumor-Associated Macrophages (TAMs), and Myeloid-Derived Suppressor Cells (MDSCs)

The PDAC TME contains abundant immunosuppressive cell types, including Tregs, TAMs, and MDSCs [22,24,37]. TAMs play a central role in the suppression of antitumor immunity, primarily exhibiting M2-like transcriptional and functional characteristics [25,36]. They promote angiogenesis and immune evasion through cytokines such as IL-10, TGF-β, and VEGF [25,36,45]. The recruitment and polarization of MDSCs are influenced by both tumor-intrinsic signaling and stromal cues [26,35,46]. Inflammatory CAFs (iCAFs) secrete IL-6 and CCL2 to attract MDSCs [39,47,48], while oncogenic KRAS signaling enhances GM-CSF production, further promoting MDSC expansion and mobilization [37,49].

Tregs also inhibit antitumor responses by releasing immunosuppressive cytokines, including TGF-β and IL-10 [45]. Collectively, these overlapping immunosuppressive processes create a highly immune-resistant TME that fundamentally limits the efficacy of ICIs [22,23,49]. Importantly, this immunosuppression varies spatially and temporally across tumor regions, enabling different tumor subclones to simultaneously evade immune surveillance through multiple mechanisms [12,20,28].

#### 2.2.2. Role of Inhibitory Cytokines: Induction of Immunotolerance via TGF-β and IL-10

Immunosuppressive cells promote immune resistance by secreting inhibitory cytokines such as TGF-β and IL-10, which collectively diminish the therapeutic effect of ICIs [21,22,23,49]. TGF-β is particularly significant in suppressing antitumor T-cell responses by preventing T-cell infiltration into the tumor parenchyma and reinforcing intrinsic resistance to checkpoint blockade [22,39]. Tregs represent a major source of TGF-β, thereby amplifying immunosuppressive signaling in the PDAC TME, and broadly reducing antitumor immune activity [45].

### 2.3. Spatial Immune Topography and Immune Exclusion

Spatial transcriptomic and multiplex imaging studies have revealed substantial heterogeneity in the immune architecture of PDAC [20,28]. Most tumors exhibit immune-desert or immune-excluded phenotypes, wherein CD8^+^ T-cells accumulate in the peritumoral regions rather than infiltrating the tumor parenchyma [20,22,24]. Truly inflamed regions with robust T-cell entry are uncommon and are typically associated with MSI-H/dMMR tumors or treatment-induced states [8,9,22]. These immune phenotypes frequently coexist within a single tumor, producing mosaic microenvironments molded by CAF density, ECM stiffness, hypoxia, and vascular collapse [20,39,44,47]. This spatial heterogeneity contributes substantially to the profound resistance of PDAC to ICIs [22,23,49].

#### Immune-Desert, Immune-Excluded, and Inflamed Phenotypes in PDAC

The immune architecture of PDAC is predominantly characterized by immune-desert and immune-excluded phenotypes, which jointly account for approximately 60–90% of cases and define PDAC as a prototypical “cold” tumor [22,23,49]. Immune-desert tumors lack tumor-specific T-cells in both the parenchyma and stroma, reflecting a failure to initiate effective antitumor immunity [21,49]. Immune-excluded tumors display abundant CD8^+^ T cells that remain confined to the peritumoral stroma due to barriers imposed by dense ECM deposition, aberrant vasculature, and exclusionary cues such as TGF-β and CXCL12 [15,32,33,47]. Truly immune-inflamed PDAC lesions are rare and are typically restricted to MSI-H/dMMR or unusually high TMB subsets [8,9,22].

A hallmark of PDAC is the presence of immune states that do not exhibit uniform, tumor-wide patterns, but instead form spatially heterogeneous, mosaic-like immune niches within the same tumor [20,28]. High-resolution spatial analyses have revealed that regions of T cell desertification, stromal confinement, and partial inflammation can coexist within a single lesion, driven by local variations in CAF density, vascular collapse, hypoxia, and metabolic gradients [20,39,40,41,47]. This intratumoral immune heterogeneity constitutes a major barrier to effective immune checkpoint blockade, and underscores the necessity for therapeutic strategies to address the spatial complexity of the PDAC immune ecosystems [22,23,49].

### 2.4. CAF-Mediated Immune Exclusion Mechanisms

CAFs are among the most abundant stromal components in PDAC and exist in multiple plastic states, including myofibroblastic CAFs (myCAFs), inflammatory CAFs (iCAFs), and antigen-presenting CAFs (apCAFs) [22,30,47]. The functional roles of CAFs are contextually heterogeneous and cannot be reduced to a single tumor-promoting axis: while certain CAF subtypes enforce immune exclusion, experimental evidence also demonstrates tumor-restraining and barrier functions that complicate simple ablative strategies (discussed in Section 2.4.1). MyCAFs produce dense ECM and reinforce immune exclusion, whereas iCAFs secrete IL-6 and CXCL12 to shape immunosuppressive myeloid phenotypes. ApCAFs have been described as expressing MHC II without canonical co-stimulatory ligands, suggesting potentially tolerogenic interactions with CD4^+^ T-cells [22,30,47]. Among CAF-mediated pathways, the CXCL12–CXCR4 axis has been implicated in retaining T-cells within stromal regions and limiting tumor infiltration [22,32,47]. Collectively, these structural and biochemical CAF-derived barriers create a major impediment to effective immunotherapy in PDAC.

#### 2.4.1. CAF Subtypes and Plasticity: myCAFs, iCAFs, apCAFs

Single-cell transcriptomic studies have identified multiple CAF subtypes in PDAC, including myCAFs, iCAFs, and apCAFs, each occupying distinct spatial domains and functional niches [22,30,47].

- myCAFs, located adjacent to tumor glands, express high levels of alpha-smooth muscle actin (αSMA) and ECM-remodeling genes, driving desmoplasia and matrix stiffening [30,32,43,44].
- iCAFs, situated more distally within the stroma, secrete IL-6, LIF, CXCL12, and other cytokines that modulate myeloid recruitment and promote immunosuppression [22,39,47,48].
- apCAFs express MHC II and CD74 but lack co-stimulatory ligands, suggesting a tolerogenic influence on CD4^+^ T-cell responses [22,30,47].

Importantly, CAFs do not represent fixed lineages, but are plastic states regulated by interleukin-1 (IL-1)/Janus kinase-signal transducer and activator of transcription 3 (IL-1/JAK–STAT3) and TGF-β/SMAD signaling, which exert opposing pressures on subtype differentiation [30,47,48]. Recent multi-omics and spatial atlases indicate an expanded continuum of CAF states and highlight frequent transitions between phenotypes depending on inflammatory, mechanical, and metabolic cues [20,28,47]. This dynamic plasticity suggests that reprogramming upstream signaling pathways (e.g., IL-1, JAK/STAT, TGF-β) may be more effective than broad fibroblast depletion in reshaping the immunosuppressive stroma in PDAC [14,16,32,39,47]. The functional complexity of CAFs extends beyond their immune-exclusionary roles and includes a paradoxically tumor-restraining dimension that has important implications for therapeutic design. Seminal work by Özdemir et al. demonstrated that genetic depletion of αSMA^+^ myofibroblasts in murine PDAC not only failed to improve outcomes but accelerated tumor progression and reduced survival, accompanied by increased immunosuppression and a more undifferentiated tumor phenotype [14]. Parallel findings by Özdemir et al. showed that stromal elements can constrain PDAC growth, and that their loss promotes a more aggressive, invasive phenotype [14]. Hedgehog pathway inhibition, which reduces desmoplastic stroma in preclinical models, produced similar paradoxical acceleration of tumor growth in genetically engineered PDAC models, suggesting that certain stromal compartments exert tumor-restraining functions [16]. These data underscore a critical conceptual tension in the field: CAFs are not uniformly tumor-promoting. The stroma simultaneously imposes physical barriers to immune infiltration and constrains tumor invasion, and indiscriminate fibroblast ablation may disrupt tumor-restraining functions while failing to restore immune access. This distinction between stromal depletion and stromal normalization or reprogramming is clinically crucial and has directly informed the failure of broad stromal depletion strategies discussed in Section 6.3. Accordingly, therapeutic strategies that selectively reprogram immunosuppressive CAF states—rather than eliminating CAFs globally—represent a more biologically rational approach [14,16,47].

#### 2.4.2. Immune Exclusion Mechanism of CAF: Building Physical Barriers and Activating the CXCL12/CXCR4 Axis

CAFs promote immune exclusion in PDAC via two major mechanisms [22,32,39,44,47]:

(1) Physical barrier formation: Excessive ECM production by CAFs, including collagen I and hyaluronic acid, creates a dense stromal barrier that restricts T-cell penetration into the tumor parenchyma and limits the intratumoral distribution of anticancer drugs [15,32,33,43,44].
(2) Chemokine-mediated T-cell repulsion: FAP^+^ CAFs can secrete CXCL12 (SDF-1), which engages CXCR4 on T-cells and contributes to their retention within stromal compartments, thereby limiting intratumoral infiltration [22,32,47]. CXCR4 inhibition is being explored as a strategy to restore T-cell entry and improve the efficacy of ICIs in PDAC [22,23]. Further, CAFs may directly suppress T-cell activity through expression of immune checkpoint ligands [22,39,47].

Collectively, these CAF-driven structural and biochemical barriers not only mold the immune-excluded architecture of PDAC, but also exert strong selective pressures on tumor subclones [22,32,39,47]. This provides a biological bridge to the subsequent section, wherein we explore how genetic and non-genetic heterogeneity emerges and co-evolves with these microenvironmental constraints [12,13,47].

## 3. Genetic, Epigenetic, and Non-Genetic Dimensions of PDAC Heterogeneity

A major determinant of PDAC resistance to ICIs is its pronounced ITH. Beyond genetic alterations, ITH comprises epigenetic, transcriptional, metabolic, and non-genetic layers that collectively shape the biological complexity and adaptive capacity of PDAC. ITH is commonly described along the genetic, non-genetic, and spatial axes, giving rise to distinct subclonal populations that coexist within the same tumor. These subclones exhibit variable phenotypes and selective fitness advantages, driving tumor evolution, therapeutic resistance, and immune evasion [12,13,50,51].

### 3.1. Genetic and Clonal Diversity

Genetic heterogeneity in PDAC originates from recurrent driver alterations in KRAS, TP53, SMAD4, and CDKN2A, as well as substantial diversity in neoantigen landscapes [50,51]. ICI responsiveness depends not only on the number, but also on the clonal architecture of neoantigens, with clonal neoantigens eliciting more durable T-cell recognition than subclonal variants [52,53,54]. PDAC is characterized by a generally low tumor mutational burden and a limited representation of high-quality clonal neoantigens, both of which constrain effective immune detection [50,51,52,53,54]. As discussed in Section 3.2, immune pressure promotes the emergence of immune-evasive subclones through the loss of antigen-presentation machinery (HLA-I and β2-microglobulin (B2M)) and disruptions in interferon signaling, reinforcing the genetic underpinnings of immune resistance [55,56,57].

#### 3.1.1. Neoantigen Properties Determining ICI Reactivity: Qualitative and Quantitative Balance of Clonal Versus Subclonal Neoantigens

The effectiveness of ICI therapy is influenced by both qualitative and quantitative characteristics of tumor neoantigens. Tumors with a high proportion of clonal neoantigens uniformly expressed across all tumor cells tend to elicit stronger and more sustained T-cell responses. In contrast, tumors enriched in subclonal neoantigens present a heterogeneous array of immune targets, diluting effective T-cell recognition and reducing ICI responsiveness [52,53,54]. Therefore, the balance between clonal and subclonal neoantigens is a key determinant of immunotherapy outcomes [52,53,54].

#### 3.1.2. Tumor Evolution and Clone Selection After Treatment

Therapeutic interventions introduce additional selective pressures that promote the emergence of resistant tumor clones. Immune-mediated selection (immunoediting) can favor the outgrowth of subclones that lose immunogenic neoantigens or acquire defects in antigen presentation [55].

Mutations in B2M disrupt stable surface expression of MHC class I, whereas JAK1/2 mutations impair interferon-γ signaling, collectively reducing tumor visibility to cytotoxic T-cells [56,57]. These alterations represent canonical mechanisms of acquired immune escape and may support the expansion of immune-evasive subclones in treatment-exposed PDAC [56,57].

Consequently, genetic and clonal heterogeneity continuously shape PDAC evolution and immune resistance throughout the therapeutic course [55,56,57]. These mechanisms carry direct therapeutic implications: the loss of B2M or JAK1/2 mutations represent actionable resistance biomarkers that may predict failure of ICI-based regimens and inform patient selection for alternative immune strategies. Conversely, tumor subclones retaining clonal neoantigens may be preferentially targeted by personalized such as the personalized mRNA neoantigen vaccine autogene cevumeran, which demonstrated neoantigen-specific T-cell responses in 50% of surgically resected PDAC patients in a Phase I trial [55,56,57]. This genetic dimension of resistance further underscores the rationale for longitudinal ctDNA monitoring (Section 5.2.2), which can capture the emergence of these immune-escape mutations in near real time, enabling adaptive therapeutic modification [58,59,60,61].

### 3.2. Epigenetic and Transcriptomic Diversity

Epigenetic remodeling is a key driver of transcriptional heterogeneity in PDAC. Alterations in enhancer activity, DNA methylation, and chromatin accessibility generate plastic transcriptional states that enable tumor cells to rapidly adapt to microenvironmental stress or therapeutic pressure [62,63]. These epigenetic alterations can promote transient drug-tolerant phenotypes and downregulate antigen presentation pathways, including the HLA-I and interferon response programs, thereby facilitating immune evasion [62,63,64,65]. Although direct causal links to ICI resistance have not been fully elucidated, growing evidence supports the central role of epigenetic plasticity in PDAC progression and treatment failure [62,63].

#### 3.2.1. Acquisition of Functional Heterogeneity Through Regulatory Plasticity of Gene Expression

PDAC cells exhibit pronounced regulatory plasticity, enabling reversible switching between transcriptional programs in response to microenvironmental cues or therapeutic stress. This non-genetic adaptability enables tumor cells to acquire survival advantages and drug-tolerant states without requiring new genetic mutations, thereby contributing to functional heterogeneity [62,63,66].

#### 3.2.2. Acquisition of Stem Cell-like Status and Drug Resistance Through Epigenetic Reprogramming

Epigenetic reprogramming drives the emergence of stem cell-like states in PDAC and promotes therapeutic resistance. This process involves changes, such as histone deacetylation, enhancer reactivation, and chromatin reorganization, enabling the formation of drug-tolerant persister (DTP) cells that evade apoptosis despite cytotoxic therapy [66,67]. These persister states may persist after treatment cessation and serve as reservoirs for the recurrence and expansion of resistant clonal populations [66,67].

#### 3.2.3. Induction of Immune Evasion: Epigenetic Inhibition of Antigen Presenting Molecules and IFN Signaling

Epigenetic remodeling directly contributes to immune evasion in PDAC. Under immune-selective pressure, tumor cells can silence the antigen-presentation machinery (HLA-I and B2M) through DNA methyltransferase (DNMT)-mediated DNA methylation and histone-modifying enzymes, while simultaneously dampening interferon-response pathways [62,63,64,65]. These changes may also increase the expression of immunosuppressive ligands such as PD-L1 and CD47 [62,63]. Such epigenetic repression patterns correlate with poor responses to ICI therapy, and spatial transcriptomic studies have linked these transcriptional alterations to immune-excluded tumor regions characterized by limited T-cell infiltration [20,22,28,62,63].

### 3.3. Non-Genetic Heterogeneity: Cellular Plasticity and Metabolic Rewiring

Non-genetic heterogeneity—including phenotypic plasticity and metabolic rewiring—emerges independently of genetic alterations and constitutes a major molecular basis for treatment resistance and recurrence in PDAC [12,13,62,63,68,69,70,71]. These non-genetic mechanisms allow tumor cells to rapidly adapt to microenvironmental stress and therapeutic pressure, supporting dynamic and reversible transitions that sustain long-term tumor persistence [62,63,68,69,70,71].

#### 3.3.1. Phenotypic Plasticity: EMT and CSC States

Phenotypic plasticity in PDAC is driven by dynamic transitions along the epithelial–mesenchymal spectrum and the acquisition of stem cell-like properties. Epithelial–mesenchymal transition (EMT) programs—regulated by TGF-β, IL-6, Wnt/β-catenin, and Notch signaling—modulate invasion, metastatic potential, and drug sensitivity [72,73,74]. Stem-like (CSC-like) states further promote resistance through cellular quiescence, metabolic rewiring, and chromatin remodeling [66,67,73]. Collectively, EMT and CSC plasticity contribute to immune evasion by reducing antigen presentation, altering cytokine outputs, and fostering heterogeneous immune-excluding niches within the TME [62,63,72,73,74].

#### 3.3.2. Metabolic Heterogeneity: A Spatially Structured, Multimodal Stromal–Tumor Metabolic Ecosystem

The desmoplastic and hypovascular architecture of PDAC establishes a highly heterogeneous metabolic landscape wherein tumor and stromal cells engage in multimodal nutrient exchange to maintain survival under extreme nutrient- and oxygen deprivation. This metabolic heterogeneity emerges through spatially distinct gradients of oxygen, glucose, amino acids, lipids, and pH, collectively creating localized metabolic niches that shape nongenetic adaptation, immune evasion, and therapeutic resistance [68,69,70,71].

PDAC cells exhibit remarkable metabolic flexibility, relying on a combination of aerobic glycolysis, oxidative phosphorylation (OXPHOS), macropinocytosis-mediated scavenging, autophagy, and exogenous nutrient uptake from surrounding stromal populations [68,69,70]. CAFs and pancreatic stellate cells serve as critical metabolic partners that supply lactate, pyruvate, alanine, acetate, and lipids [71]. These metabolic interactions have expanded beyond the classic reverse Warburg framework to include amino-acid shuttles, autophagy-dependent nutrient release, and broader stromal–tumor nutrient exchange mechanisms [68,69,70,71].

Experimental studies suggest that these nutrient-sharing pathways are spatially organized and occur in region-specific patterns, with hypoxic, collagen-dense regions supporting lactate-rich microdomains, whereas tumor-distal PSC clusters secrete alanine and other alternative carbon sources [71]. Such metabolic heterogeneity directly impacts immune function. Lactate and kynurenine suppress T-cell activation, promote T-cell dysfunction, and foster regulatory myeloid phenotypes, thereby contributing to the establishment and maintenance of immune-excluded states [41,42]. Moreover, histone lactylation has been described as a potential mechanism by which lactate may influence the chromatin landscape; however, this phenomenon has been characterized primarily in non-PDAC systems, and whether it operates as a functionally significant pathway in PDAC remains speculative [75].

Collectively, metabolic heterogeneity constitutes a major non-genetic axis that enables PDAC cells to withstand microenvironmental stress, sustain subclonal diversity under selective pressure, and evade immune surveillance. This multimodal stromal–tumor metabolic network provides a mechanistic basis for the persistent resistance to both cytotoxic therapy and immune checkpoint blockade observed in PDAC [68,69,70,71,75].

Taken together, the genetic, epigenetic, transcriptional, and metabolic forms of ITH do not operate in isolation. Rather, they interact dynamically with their surrounding microenvironments. Therefore, the subsequent section examines how these heterogeneous tumor cells reciprocally mold, and are molded, by the TME to generate self-reinforcing circuits of immune resistance [62,63,68,69,70,71,75].

## 4. Dynamic and Reciprocal Crosstalk Between ITH and TME

### 4.1. Reciprocal Shaping of Tumor Cells and Microenvironment

**Figure 1:**
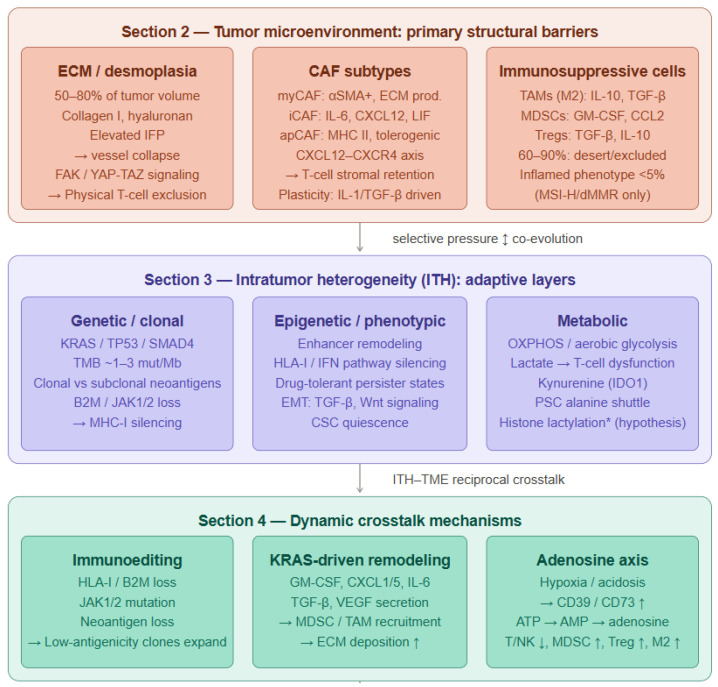
**Integrated mechanistic framework of intratumor heterogeneity–tumor microenvironment co-evolution in pancreatic ductal adenocarcinoma.** This schematic depicts three hierarchical axes of immune resistance in PDAC. Section 2 (**top**, coral) illustrates the primary structural barriers of the TME: desmoplastic ECM (50–80% of tumor volume; elevated IFP, vessel collapse, FAK/YAP-TAZ signaling, physical T-cell exclusion), CAF subtypes (myCAF, iCAF, apCAF; CXCL12–CXCR4-mediated T-cell retention; IL-1/TGF-β-driven plasticity), and immunosuppressive cell populations (TAMs, MDSCs, Tregs) that impose an immune-desert or immune-excluded phenotype in 60–90% of PDAC lesions (<5% inflamed; MSI-H/dMMR only). Section 3 (**middle**, purple) depicts adaptive ITH layers: genetic/clonal heterogeneity (KRAS/TP53/SMAD4 mutations, TMB ~1–3 mut/Mb, clonal vs. subclonal neoantigens, B2M/JAK1/2 loss → MHC-I silencing), epigenetic/phenotypic plasticity (enhancer remodeling, HLA-I/IFN pathway silencing, EMT via TGF-β/Wnt signaling, CSC quiescence), and metabolic rewiring (OXPHOS/glycolysis switching, lactate-mediated T-cell dysfunction, IDO1/kynurenine axis, PSC alanine shuttle, histone lactylation *). Section 4 (**bottom**, teal) summarizes ITH–TME reciprocal crosstalk: immunoediting (HLA-I/B2M loss, neoantigen loss → low-antigenicity clone expansion), KRAS-driven remodeling (GM-CSF, CXCL1/5, IL-6, TGF-β, VEGF → MDSC/TAM recruitment, ECM deposition), and the adenosine axis (hypoxia/acidosis → CD39/CD73 upregulation → ATP → AMP → adenosine → T/NK cell suppression, MDSC ↑, Treg ↑, M2 ↑). Arrows indicate selective pressure and co-evolutionary dynamics between ITH and the TME. Abbreviations: CAF, cancer-associated fibroblast; CSC, cancer stem cell; ECM, extracellular matrix; EMT, epithelial–mesenchymal transition; FAK, focal adhesion kinase; IDO1, indoleamine 2,3-dioxygenase 1; IFP, interstitial fluid pressure; ITH, intratumor heterogeneity; MDSC, myeloid-derived suppressor cell; MHC-I, major histocompatibility complex class I; MSI-H, microsatellite instability-high; dMMR, mismatch repair-deficient; OXPHOS, oxidative phosphorylation; PDAC, pancreatic ductal adenocarcinoma; PSC, pancreatic stellate cell; TAM, tumor-associated macrophage; TGF-β, transforming growth factor-beta; TMB, tumor mutational burden; TME, tumor microenvironment; Treg, regulatory T cell. * Histone lactylation is presented as a conceptual hypothesis; direct causal validation in PDAC remains lacking.

ITH and the TME engage in reciprocal feedback loops that drive adaptive tumor evolution and therapeutic resistance. These dynamic interactions continuously reshape both the tumor cells and their surrounding stroma, thereby reinforcing phenotypic diversity and immune evasion [76,77,78,79] (Figure 1).

#### 4.1.1. Selective Survival of Immune-Evasive Clones Under Microenvironmental Stress

Hypoxia, nutrient scarcity, stromal stiffness, and chronic inflammation generate strong selective pressures in PDAC. Tumor subclones adapt through metabolic rewiring, the activation of antioxidant programs, and the downregulation of antigen-presentation pathways [76,77]. Spatial transcriptomic studies have indicated that hypoxic niches activate HIF-1α-dependent metabolic programs, while maintaining OXPHOS activity in certain contexts [28,40,76]. NRF2-driven antioxidant defenses further enhance survival under oxidative and therapeutic stress, facilitating the expansion of highly tolerant immune-evasive subclones [76].

#### 4.1.2. Oncogenic Signaling–Driven TME Remodeling

Oncogenic pathways—particularly mutant KRAS signaling—play a central role in remodeling stromal and immune components in PDAC. KRAS-driven tumor cells secrete cytokines such as IL-6, CXCL1, CXCL5, GM-CSF, and TGF-β, which activate CAFs, promote angiogenic and fibrotic programs, and recruit immunosuppressive myeloid populations [77,78]. These interactions are consistent with a dynamically coupled relationship between tumor clones and the TME, reinforcing immune resistance and enabling the emergence of highly tolerant subclonal lineages [77,78]. Whether this coupling constitutes a formally co-evolving circuit in the strict evolutionary sense remains to be demonstrated in longitudinal PDAC-specific datasets.

### 4.2. Immune-Driven Selection of Low-Antigenicity Subclones

Immune surveillance exerts selective pressure that promotes the expansion of subclones with low antigenicity. These include variants that may lose immunogenic neoantigens, downregulate HLA-I or B2M, or acquire JAK1/2 alterations that impair interferon (IFN) signaling. This immune-editing process reduces T-cell recognition and supports the emergence of immune-evasive phenotypes that drive resistance to immune checkpoint blockade [55,56,57].

### 4.3. Interclonal Cooperation Model

PDAC represents a complex multicellular ecosystem in which diverse cancer subclones and stromal elements coexist. These subclones compete, yet collectively reinforce immunosuppression and therapeutic resistance. Interclonal cooperation helps maintain ITH and enhances the overall robustness and adaptability of the tumor [79].

#### 4.3.1. Public-Goods-Driven Non-Cell-Autonomous Cooperation

In PDAC, interclonal cooperation has been substantiated by several lines of PDAC-specific evidence. Single-cell RNA-sequencing studies in PDAC [80] have identified transcriptionally distinct malignant subclone populations within the same tumor that exhibit differential expression of growth factors and cytokines, consistent with a public-goods–producing hierarchy [80]. Spatially resolved analyses [20,28] further demonstrate that cytokine gradients—including GM-CSF and IL-6 produced by KRAS-high subclones—spatially recruit immunosuppressive myeloid populations preferentially toward hypoxic, desmoplastic niches, thereby indirectly shielding neighboring low-KRAS subclones from immune attack [20,28]. Additionally, CAF-derived extracellular vesicles containing miR-106b confer gemcitabine resistance not only to recipient tumor cells but also to metabolically distinct subclones incapable of intrinsic resistance, thereby expanding collective chemoresistance across the clonal hierarchy [81,82]. Collectively, these PDAC-specific data support the concept that interclonal cooperation is not merely theoretical but a spatially structured, clinically relevant mechanism that sustains ITH under therapeutic pressure [79,81,82].

#### 4.3.2. Metabolic Crosstalk

Metabolic crosstalk in PDAC represents a streamlined set of high-impact, non-cell-autonomous nutrient-sharing interactions that enhance tumor adaptability under nutrient-poor conditions. Rather than encompassing the full spectrum of metabolic heterogeneity, this section focuses on two representative and well-characterized modes of stromal–tumor metabolic support.

(1) PSC-Derived Alanine Shuttle Activated PSCs differentiate into CAFs and secrete alanine via autophagy. PSC-derived alanine serves as an efficient alternative carbon source that fuels the tricarboxylic acid (TCA) cycle in PDAC cells, thereby supporting biomass synthesis and mitochondrial metabolism during glucose scarcity. The alanine shuttle exemplifies how stromal cells buffer metabolic stress and confer survival advantages to tumor cells within hypovascular niches [71].
(2) Extracellular Vesicle-Mediated Metabolite and Cargo Transfer CAFs release extracellular vesicles, particularly exosomes, that can induce context-dependent transcriptional programs linked to cellular metabolic states [62,63,83,84]. Extracellular vesicles also transport functional cargo, including microRNAs and proteins. For example, miR-106b targets TP53INP1, which promotes chemoresistance and metabolic reprogramming. Through extracellular vesicle-mediated nutrient and signal transfer, stromal cells enhance PDAC survival, invasiveness, and resistance to gemcitabine and other cytotoxic agents [81,82].

**Figure 2:**
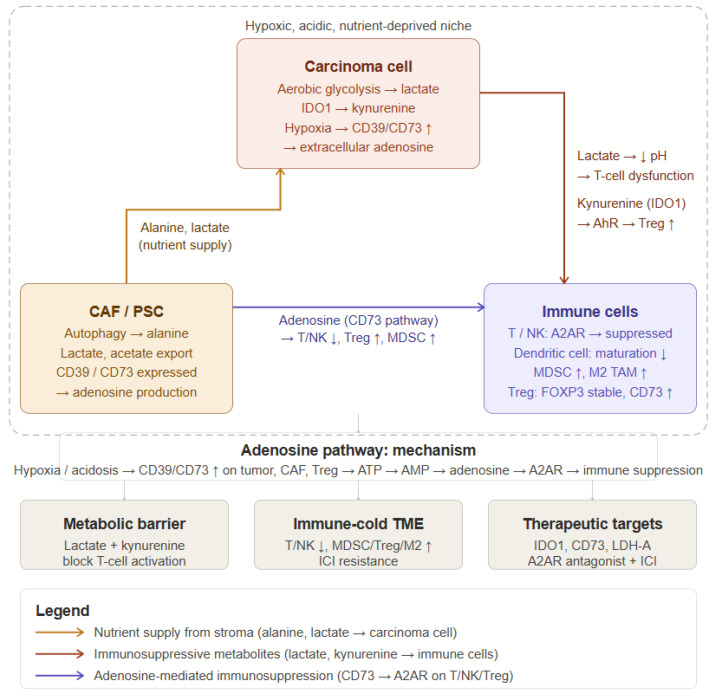
**Metabolic crosstalk between carcinoma cells, cancer-associated fibroblasts, and immune cells in the pancreatic ductal adenocarcinoma tumor microenvironment.** This schematic illustrates the bidirectional metabolic interactions among the three principal cellular components of the PDAC TME within a shared hypoxic, acidic, and nutrient-deprived niche. Carcinoma cells (coral) undergo aerobic glycolysis generating lactate, which lowers extracellular pH and drives T-cell dysfunction; they also upregulate IDO1 to catabolize tryptophan to kynurenine, which activates AhR to promote Treg differentiation. Under hypoxia, carcinoma cells further upregulate CD39 and CD73, driving extracellular adenosine accumulation. CAF/PSC (amber) supply alanine and lactate as nutrients to carcinoma cells via autophagy, and export lactate and acetate while co-expressing CD39/CD73 to amplify adenosine production within the TME. Immune cells (purple) are the principal targets of this metabolic suppression: T and NK cells are silenced via A2AR signaling; dendritic cell maturation is impaired; MDSCs and M2-polarized TAMs are expanded; and Tregs are stabilized through FOXP3 maintenance and CD73 co-expression. The adenosine pathway mechanism strip depicts the biochemical sequence: hypoxia and acidosis drive CD39/CD73 upregulation on tumor cells, CAFs, and Tregs, sequentially hydrolyzing extracellular ATP to AMP and then to adenosine, which signals through A2AR to suppress antitumor immunity. The three outcome boxes summarize functional consequences: a metabolic barrier (lactate and kynurenine blocking T-cell activation), an immune-cold TME (T/NK depletion, MDSC/Treg/M2 enrichment, ICI resistance), and therapeutic targets (IDO1, CD73, LDH-A inhibition; A2AR antagonism combined with ICI). Arrow colors indicate the direction and nature of intercellular communication: amber arrows, nutrient supply from stroma to carcinoma cells (alanine, lactate); red arrows, immunosuppressive metabolite release from carcinoma cells to immune cells (lactate, kynurenine); purple arrows, adenosine-mediated immunosuppression via the CD73 pathway targeting T/NK cells and Tregs. Abbreviations: A2AR, adenosine A2A receptor; AhR, aryl hydrocarbon receptor; AMP, adenosine monophosphate; ATP, adenosine triphosphate; CAF, cancer-associated fibroblast; IDO1, indoleamine 2,3-dioxygenase 1; ICI, immune checkpoint inhibitor; LDH-A, lactate dehydrogenase A; MDSC, myeloid-derived suppressor cell; PDAC, pancreatic ductal adenocarcinoma; PSC, pancreatic stellate cell; TAM, tumor-associated macrophage; TME, tumor microenvironment; Treg, regulatory T cell.

Beyond these two representative modes, emerging evidence highlights a broader metabolic interaction network between carcinoma cells, CAFs, and immune cells that is central to PDAC immune suppression (Figure 2). CAF-derived lactate is exported via monocarboxylate transporters (MCT1/4) and taken up by tumor cells to fuel oxidative phosphorylation, while simultaneously suppressing cytotoxic T-cell function by lowering extracellular pH and impairing TCR signaling [41,71]. Concurrently, IDO1-expressing tumor cells and MDSCs catabolize tryptophan to kynurenine, which activates the aryl hydrocarbon receptor (AhR) in T cells to promote Treg differentiation and suppress Teff function [42]. Under hypoxia, CD39 and CD73 expressed on tumor cells, CAFs, and Tregs sequentially hydrolyze extracellular ATP to immunosuppressive adenosine, which acts on A2A receptors on T and NK cells to broadly suppress effector function—as detailed in Section 6.3.2 [85]. These metabolic exchanges collectively create an immunometabolic barrier that reinforces immune exclusion independent of, and in concert with, the physical barriers imposed by the desmoplastic stroma. The metabolic interactions between carcinoma cells, CAFs/PSCs, and immune cells that collectively establish immunosuppressive conditions within the PDAC TME are schematically illustrated in Figure 2.

Together, these simplified representative mechanisms illustrate how selective stromal–tumor metabolic partnerships sustain tumor fitness and complement the broader, spatially structured metabolic heterogeneity described in Section 3.3.2, thereby reinforcing ITH and driving therapeutic resistance in PDAC [41,42,71,81,82,85].

### 4.4. ECM–Metabolic–Epigenetic Coupling as a Driver of PDAC Plasticity

In PDAC, spatially specialized metabolic niches may hypothetically link metabolic states to epigenetic regulation. Metabolites such as acetyl-CoA, α-KG, and lactate are known to modulate chromatin-modifying enzymes in various cancer contexts; however, whether these interactions constitute a causally operative mechanism specifically in PDAC remains unestablished. The available evidence is largely derived from in vitro systems or non-PDAC tumor models, and direct experimental validation in PDAC is lacking. Therefore, the concept of metabolic–epigenetic coupling in PDAC should be regarded as a working hypothesis rather than a confirmed mechanistic axis, and should be interpreted with appropriate caution pending PDAC-specific in vivo validation. Therefore, while metabolite-driven histone modification is a compelling conceptual framework, the current PDAC data should be regarded as suggestive rather than definitive. Although succinate/fumarate-mediated dioxygenase inhibition is not well-documented in PDAC, hypoxia-associated metabolic shifts are likely to participate in similar epigenetic suppression. This metabolic–epigenetic axis enables the dynamic adaptation of PDAC subclones and contributes to therapeutic resistance [83,84].

#### 4.4.1. Metabolite–Chromatin Axis

Metabolites such as acetyl-CoA, α-ketoglutarate (α-KG), and 2-hydroxyglutarate (2-HG), affect the activity of chromatin-modifying enzymes, linking the metabolic state of cells to transcriptional regulation. In this process, tumor cells reversibly reorganize gene expression patterns according to energy and redox conditions, creating a clone-specific transcriptional program [83,84].

#### 4.4.2. Histone Modification by Lactate and Acetate

CAF-derived lactate and acetate may influence chromatin remodeling through distinct metabolite-sensitive epigenetic mechanisms [75,83,84]. In particular, lactate has been linked to histone lactylation, whereas acetate contributes to acetyl-CoA–dependent histone acetylation programs, thereby reshaping transcriptional output in tumor cells [75,83,84]. Consequently, these metabolic–epigenetic interactions form a mechanistic link between metabolic gradients within the TME and clone-specific transcriptional programs [62,63,75,83,84].

#### 4.4.3. Hypoxia-Associated Metabolites and Epigenetic Suppression

While succinate- or fumarate-mediated inhibition of α-KG-dependent dioxygenases is well established in SDH/IDH-mutant tumors, similar metabolic–epigenetic coupling has not been experimentally validated in PDAC and remains a theoretical possibility requiring definitive investigation. In summary, the TME–metabolic–epigenetic axis of PDAC enables the plastic evolution of tumors by directly linking cell-level energy flows with chromatin states. Disruption of this metabolic–epigenetic axis may contribute to immune avoidance, drug resistance, and the maintenance of clonal diversity in PDAC and therefore represents a potential target for future integrative metabolic–epigenetic therapeutic strategies [62,63,75,83,84].

Because these ITH–TME feedback loops operate in spatially organized and temporally evolving patterns, resolving them requires analytical tools capable of capturing interactions at single-cell and spatial resolutions. Accordingly, the subsequent section introduces recently developed multi-omics and spatial technologies that enable quantitative mapping of these complex ecological dynamics in PDAC [28,62,63].

## 5. Advanced Methodologies for Deciphering ITH–TME Dynamics

**Figure 3:**
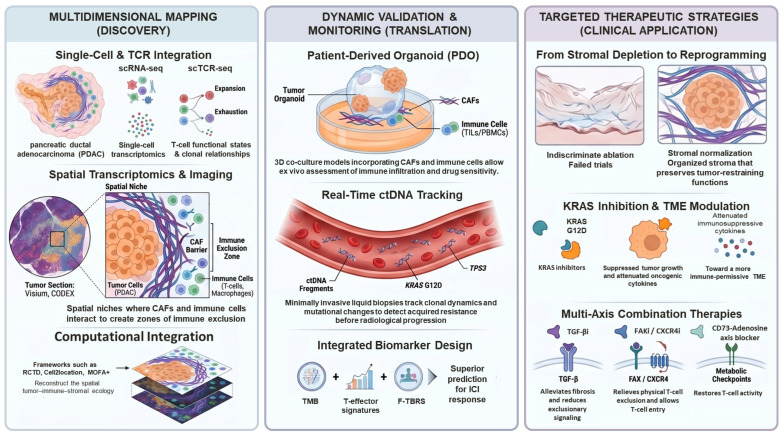
**Precision oncology framework for PDAC: From spatiotemporal mapping to targeted therapeutic strategies.** The figure illustrates a three-stage framework that links multidimensional tumor profiling to rational therapeutic strategies in pancreatic ductal adenocarcinoma (PDAC). Stage 1 (Discovery) focuses on multidimensional mapping of intratumoral heterogeneity and the tumor microenvironment using single-cell RNA sequencing and TCR sequencing to define T-cell functional states and clonal relationships, spatial transcriptomics and multiplex imaging to delineate CAF-rich immune exclusion niches, and computational integration tools (e.g., RCTD, Cell2location, MOFA+) to reconstruct the spatial tumor–immune–stromal “ecology.” Stage 2 (Translation) emphasizes dynamic validation and monitoring through patient-derived organoid (PDO) co-culture systems incorporating CAFs and immune cells to assess ex vivo immune infiltration and drug sensitivity, together with longitudinal circulating tumor DNA (ctDNA) tracking to monitor clonal evolution and emergent resistance (e.g., KRAS and TP53 mutations). These data streams are integrated into composite biomarker models that combine tumor mutational burden (TMB), T-effector (Teff) signatures, and fibroblast TGF-β response signatures (F-TBRS) to improve prediction of ICI responsiveness. Stage 3 (Clinical Application) highlights targeted therapeutic strategies informed by these datasets, illustrating the shift from indiscriminate stromal depletion (e.g., failed PEGPH20-based approaches) toward stromal normalization and reprogramming; KRAS-targeted approaches (such as KRAS G12D and pan-RAS inhibitors) aimed at attenuating oncogenic signaling and partially restoring an immune-permissive TME; and multi-axis combination regimens that concurrently modulate TGF-β signaling, FAK/CXCR4-mediated exclusion, and metabolic checkpoints, as well as DNA repair pathways (e.g., PARP inhibitor plus ICI combinations). Together, this framework proposes a path from high-resolution spatiotemporal mapping of PDAC ecosystems to precision immuno-oncology strategies tailored to individual patients.

To comprehensively understand the therapeutic resistance and poor prognosis of PDAC, it is crucial to elucidate, with high precision, the dynamic interplay between ITH and the TME. Traditional bulk-based analyses have inherent limitations in resolving single-cell-level diversity and interaction networks within tumors. Recent advancements in single-cell and spatial omics technologies have provided unprecedented tools to explore the complex ecosystem of PDAC in a spatiotemporal manner [20,28,83,84] (Figure 3).

### 5.1. Single-Cell and Spatial Omics Approaches

Recent advances in genomics have been driven by the integration of single-cell technologies with spatial profiling approaches. These approaches enable the detailed characterization of cellular populations, functional states, and signaling axes that constitute the immunosuppressive ecosystem of PDAC, thereby facilitating the identification of novel therapeutic targets and interaction pathways [20,28,80].

#### 5.1.1. Single-Cell RNA Sequencing (scRNA-Seq) and Adaptive Immune Receptor Repertoire (AIRR/scTCR-Seq) Analysis

Single-cell transcriptomic profiling (scRNA-seq) facilitates precise characterization of the cellular composition within the TME, including malignant subclones, fibroblast subtypes, and diverse immune cell populations [28,81]. When integrated with single-cell T-cell receptor sequencing (scTCR-seq), it is possible to directly link the functional states of T-cells (as defined by gene expression programs) to their TCR sequence-based clonal relationships [82]. Such integrative approaches provide critical information for interpreting spatial and functional variations in immune responses by tracking clonal expansion and state transitions of individual T-cell clones within the TME.

A representative example is the STARTRAC algorithm, which integrates scRNA-seq and TCR data to compute indices, such as clonal expansion (STARTRAC-expa) and migratory potential (STARTRAC-migr), for specific T-cell subsets [80]. These metrics enable the quantitative dissection of how T-cell differentiation trajectories and functional adaptation are shaped by immune pressure and clonal selection.

#### 5.1.2. Spatial Transcriptomics (ST) and Imaging Mass Cytometry (IMC/CODEX)

Conventional single-cell technologies inevitably lose their positional context during tissue dissociation, limiting the interpretation of the spatial organization and microanatomical significance of cell–cell interactions. To address this challenge, spatial-omics technologies that preserve spatial resolution have become indispensable [86].

(1) Spatial Transcriptomics

Technologies such as Visium (10× Genomics), Slide-seqV2, and Stereo-seq capture spatially resolved gene expression profiles within tissue sections [86,87,88]. In PDAC, spatial transcriptomics enables high-resolution mapping of tumor subclusters and associated spatial niches [28,29]. Studies have reported that distinct CAF subtypes, including myCAFs and iCAFs, as well as immune cells, occupy segregated spatial domains that correlate with immune exclusion [20,28]. Specifically, tumor-distal niches enriched in iCAFs and immune cells have been proposed as spatial microenvironments closely associated with impaired T-cell infiltration and immune exclusion [20,28].

(2) Imaging Mass Cytometry (IMC/CODEX)

High-dimensional imaging platforms such as CyTOF-based IMC, CODEX, and multiplex fluorescence IHC (mfIHC) employ metal-conjugated antibodies or multiplexed fluorescent labels to visualize protein expression patterns within the intact tissue architecture [89,90]. These technologies facilitate the simultaneous assessment of the spatial distribution and proximity of multiple immune cell subsets, including T-cells, tumor-associated macrophages, and myeloid-derived suppressor cells [89,90]. Such spatially resolved visualization is essential for understanding location-dependent immune cell functional states and uncovering the structural constraints that shape immune networks within the TME.

#### 5.1.3. Computational Integration Frameworks

The integration of single-cell and spatial data is crucial for reconstructing the spatial ecology of PDAC. By mapping cell identities defined by scRNA-seq onto spatial transcriptomics data, both the cellular composition and spatial distribution of specific cell types across tumor regions can be inferred [89,90,91,92].

Key algorithms and frameworks, such as RCTD and Cell2location, integrate single-cell expression profiles with spatial transcriptomics data to quantitatively infer cell-type-specific spatial localization [89,90]. Seurat-based integration pipelines, MOFA+ as a multi-omics integration framework, and graph-based spatial models, such as SpaGCN, are widely employed to integrate heterogeneous omics and imaging datasets, normalize spatial transcriptomic data, and define spatial domains or niches [91,92,93].

More recently, AI-assisted spatial omics frameworks have shown potential for modeling patient-specific immune infiltration patterns and informing therapeutic response prediction [94], thereby providing a conceptual basis for the future development of patient-tailored digital twin models. Collectively, these computational integration approaches are becoming indispensable tools for dissecting immune exclusion zones, CAF–immune cell interaction axes, and spatially organized ITH–TME feedback mechanisms in PDAC [89,90,91,92,93,94].

Despite the high-resolution insights provided by these spatial multi-omics frameworks, several barriers to clinical scalability remain. The high cost of sequencing, the lack of standardized computational pipelines for cross-center data integration, and the requirement for fresh-frozen tissue in many high-plex platforms limit their routine use in clinical practice. Future efforts will need to prioritize developing cost-effective, FFPE-compatible spatial assays to enable large-scale, clinically actionable patient stratification.

### 5.2. Functional Modeling and Longitudinal Monitoring

The interaction between ITH and the TME is not a static relationship but a dynamic process that continuously evolves under therapeutic pressure and over time. Consequently, experimental systems that recapitulate these interactions and technologies that enable noninvasive monitoring of tumor evolution during treatment are becoming increasingly important in PDAC research [95].

#### 5.2.1. Patient-Derived Organoids (PDOs) and Immune Co-Culture Systems

(1) Recapitulation of Patient-Specific Tumor Epithelial Features

Patient-derived organoids (PDOs) are three-dimensional cultures obtained from tumor epithelial cells. Compared with conventional two-dimensional cell lines, PDOs more faithfully preserve the genetic alterations and epithelial features (e.g., KRAS mutations) and epithelial morphological and molecular characteristics of the primary tumor [95,96]. More recently, efforts have been made to incorporate non-epithelial components such as CAFs, immune cells, and tailored ECM compositions into composite models to better approximate stromal and microenvironmental features of PDAC [97,98].

(2) Assessment of Immune Responsiveness and Immunotherapy Efficacy

Since the efficacy of ICIs critically depends on T-cell interactions, PDO-based immune co-culture models have emerged as innovative experimental platforms that incorporate autologous immune components [99]. In these systems, patient-derived peripheral blood mononuclear cells (PBMCs) or tumor-infiltrating lymphocytes (TILs) are co-cultured with PDOs to reconstruct key aspects of the TME in vitro, enabling the direct assessment of immune cell activation, infiltration, and tumor cell killing [58,99].

(3) Prediction of Patient-Specific Drug Responses

PDO–immune co-culture systems allow the experimental evaluation of patient-specific immune-mediated tumor killing and can serve as platforms to predict responses to ICIs and combination regimens [58].

(4) Modeling Desmoplastic Microenvironments and Drug Resistance

Recently, engineered organoid–stroma hybrid models and microfluidic platforms have been developed to incorporate stromal components and modulate matrix stiffness and ECM composition [98]. These systems aim to better approximate desmoplastic microenvironmental features and provide refined experimental models for investigating drug penetration barriers and chemoresistance mechanisms [98]. Despite these advantages, current PDO-based immune co-culture models remain reductionist systems that incompletely capture the full vascular, immune, stromal, and temporal complexity of the in vivo PDAC ecosystem, which may limit their predictive translation to clinical therapeutic responses.

#### 5.2.2. Circulating Tumor DNA (ctDNA) Analysis

To comprehend the evolutionary dynamics of PDAC, it is essential to track the temporal changes occurring throughout the course of treatment. Under therapeutic selective pressure, subclonal populations may expand or diminish, and longitudinal tracking provides a means to reconstruct ITH in near real-time [58].

(1) ctDNA as a Minimally Invasive, Real-Time Diagnostic Tool

Circulating tumor DNA (ctDNA) comprises tumor-derived DNA fragments released into the bloodstream upon tumor cell death and can be obtained via minimally invasive blood-based liquid biopsy [58]. ctDNA analysis offers a clinically powerful tool for monitoring the genetic evolution of tumors and elucidating the mechanisms of drug resistance in near real time [59].

(2) Tracking Clonal Dynamics and Mutational Changes

ctDNAs reflect the composite genetic information of multiple subclonal populations originating from both primary tumors and metastatic lesions [59,60]. Longitudinal next-generation sequencing profiling, employing either targeted panels or exome-level sequencing of key driver genes, such as KRAS, TP53, SMAD4, and CDKN2A, enables quantitative assessment of temporal changes in somatic mutations and restructuring of clonal architecture in individual patients [60].

(3) Clinical Utility and Predictive Value

ctDNA levels and mutational patterns reflect tumor burden and the tempo of tumor evolution, and numerous studies have demonstrated that ctDNA dynamics can predict radiological disease progression or relapse [59,60,61]. Such real-time monitoring facilitates the early detection of acquired resistance to targeted therapies and ICIs and supports adaptive therapeutic decision-making by enabling timely modification or intensification of treatment strategies [59,60,61].

### 5.3. Clinical Feasibility, Logistics, and Practical Bottlenecks of Multimodal Integration

While the integration of longitudinal ctDNA tracking, spatial transcriptomics, and patient-derived organoid (PDO) drug screening presents a conceptually attractive framework for precision immuno-oncology, significant practical and logistical bottlenecks currently limit its routine translation to clinical reality [20,28,58,59,60,61].

(1) Feasibility of Repeated Spatial Transcriptomics:

Performing serial, high-plex spatial profiling (e.g., Visium, CODEX) to monitor ITH–TME co-evolution is currently unfeasible in standard clinical care. Unlike hematological malignancies, obtaining repeated tumor tissue from PDAC is deeply constrained by anatomical barriers, procedural risks (e.g., pancreatitis, bleeding), and the profound pain or exhaustion of metastatic patients. Furthermore, the extreme tissue heterogeneity of PDAC means that single-core re-biopsies are highly prone to sampling bias, rendering longitudinal spatial comparison technically unreliable and economically prohibitive for routine screening.

(2) Realism of Serial ctDNA-Guided Adaptive Immunotherapy:

Tracking clonal dynamics via circulating tumor DNA (ctDNA) offers a minimally invasive alternative for real-time monitoring and is increasingly being incorporated into observational and interventional PDAC studies [58,59,60,61]. However, liquid biopsies in PDAC suffer from inherently low shedding dynamics compared to other solid tumors like colorectal cancer or melanoma, often leading to false-negative results in early-stage or locally advanced disease [60,61]. Adjusting complex multi-axis immunotherapies dynamically based on subclonal genomic shifts remains a future aspiration rather than a current clinical tool, as validated adaptive clinical trial designs and standardized computational pipelines for real-time data integration are completely lacking.

(3) Clinical Cost and Toxicity Burden of Multi-Axis Combinations:

Simultaneously targeting oncogenic pathways (KRAS), stromal architecture (TGF-β/FAK), and immunometabolic checkpoints (CD73/IDO1) exponentially escalates both financial cost and systemic toxicity. Early-phase trials combining immune checkpoint inhibitors with stromal or myeloid-targeting agents have frequently observed severe immune-related adverse events (irAEs), hepatotoxicity, or systemic fibroproliferative complications. Navigating the narrow therapeutic window of these combined modalities requires rigorous dose-escalation protocols and patient-specific biomarker enrichment to avoid compromising patient safety for theoretical synergy [100,101].

## 6. ITH–TME-Guided Combination Therapeutic Strategies

PDAC exhibits profound ITH and a strongly immunosuppressive TME, which together contribute to very low response rates to ICI monotherapy. This biological complexity underlies the classification of PDAC as an immunologically “cold” tumor and serves as a major driver of therapeutic resistance. Consequently, the focus of recent clinical research has shifted from single-target approaches toward the development of rational combination therapies designed to simultaneously block multiple resistance axes [102,103] (Figure 2). However, despite strong mechanistic rationale and encouraging preclinical findings, most combination immunotherapy strategies in PDAC have thus far produced only modest clinical benefit in early-phase trials. This translational gap reflects the extreme biological complexity of PDAC, substantial interpatient heterogeneity, limited intra-tumoral drug penetration caused by desmoplasia, and the absence of validated biomarkers capable of accurately stratifying patients for specific therapeutic combinations [104].

### 6.1. Clonal Diversity-Based ICI Response Prediction and Integrated Biomarker Design

The pronounced spatial and temporal heterogeneity of PDAC results in substantial sampling bias when relying on single biopsies and conventional bulk sequencing. To overcome these limitations and more accurately predict ICI responsiveness, there is a growing need to develop multiparametric biomarkers that integrate information on tumor clonal diversity, T-cell functional states, and immunosuppressive signaling within the TME [105]. Across several solid tumor cohorts, models that combine tumor mutational burden (TMB), CD8^+^ T effector (Teff) signatures, and fibroblast TGF-β response signatures (F-TBRS) or related TGF-β–driven stromal signatures have demonstrated superior predictive performance for ICI response compared to individual factors alone [100,106]. Such integrated frameworks simultaneously capture the interplay between intratumoral clonal diversity and immunosuppressive stromal/immune signals and are now being explored as promising approaches for PDAC [106]. Ongoing studies are attempting to adapt and validate these composite biomarkers using PDAC-specific datasets. Ultimately, the successful implementation of ICI therapy in PDAC will require the integrated analysis of high-resolution, spatially resolved profiling of ITH and TME and the establishment of a precision immunoprofiling framework that provides robust prognostic and predictive stratification for individual patients [100,105,106]. A major unresolved challenge is that clinically relevant immune phenotypes in PDAC are highly dynamic and spatially heterogeneous, making reliable patient stratification difficult when based on single-site biopsies or static molecular profiling alone [107,108]. Furthermore, biomarkers associated with ICI responsiveness, including MSI-H/dMMR and high-TMB status, are exceedingly rare in PDAC [47,107].

Consequently, clinically actionable biomarker frameworks will likely require longitudinal and spatially integrated approaches combining ctDNA, spatial omics, and functional ex vivo modeling.

### 6.2. Targeting Tumor-Intrinsic Clones and Drivers

Overcoming therapeutic resistance in PDAC requires modulation of the TME and direct targeting of tumor-intrinsic factors that drive immune evasion.

#### 6.2.1. Reprogramming the Immunosuppressive TME via KRAS Inhibition

KRAS is the canonical oncogenic driver of PDAC, with activating mutations present in approximately 90% of PDAC cases. KRAS signaling promotes tumor cell survival and proliferation, as well as contributing to the establishment of an immunosuppressive TME [77,109]. Oncogenic KRAS enhances the production of cytokines and growth factors such as GM-CSF, CXCL8 (IL-8), VEGF, and TGF-β, thereby recruiting MDSCs and TAMs, and reinforcing immunosuppressive networks [77,109,110]. Consequently, inhibition of the KRAS pathway has the potential to suppress tumor cell–intrinsic growth signals, while simultaneously attenuating the generation of immunosuppressive mediators, and reprogramming the TME. MRTX1133, a selective KRAS G12D inhibitor, has shown robust preclinical activity in murine PDAC models and evidence of TME modulation. In preclinical studies, MRTX1133 monotherapy inhibited tumor growth, whereas combination therapy with anti-PD-1 or anti-CTLA-4 antibodies led to CD8^+^ T-cell–dependent tumor regression and prolonged survival [111]. In parallel, RMC-6236, an orally administered pan-RAS inhibitor, has shown early clinical activity in KRAS-mutant PDAC in phase 1/Ib studies and has advanced into phase 3 evaluation (RASolute 302) in metastatic PDAC [112]. These findings suggest that targeting KRAS can convert the immune-refractory TME of PDAC into a more immune-permissible state and potentially enhance ICI-based combination strategies. However, whether these effects translate into durable benefit in patients with heavily desmoplastic human tumors remains uncertain, and adaptive bypass signaling, clonal resistance, and mutation-specific constraints continue to limit broader applicability [113,114].

#### 6.2.2. Combination Strategies with PARP Inhibitors and ICIs

Defects in DNA damage repair (DDR) represent a molecular vulnerability in a subset of patients with PDAC, and PARP inhibitors (PARPi) have become established targeted agents particularly in tumors harboring BRCA1/2 and related homologous recombination repair mutations [115]. However, the durability of responses to PARPi monotherapy is often limited, and in some settings, PARP inhibition may induce the compensatory activation of immune-evasive feedback pathways, including the upregulation of PD-L1 and immunosuppressive cytokines [116]. This has led to the proposal of combination strategies that incorporate PARPi and ICIs. Larger, molecularly stratified trials are required before the PARP–ICI combination can be considered a validated therapeutic strategy for PDAC. These data suggest that immune checkpoint modulation may provide benefit in selected molecular subgroups of PDAC, and that dual targeting of the DDR axis and immune checkpoints warrants further evaluation in molecularly stratified settings [115,116].

### 6.3. Strategies for Remodeling the Immunosuppressive TME

The transition toward TME-modulating strategies was catalyzed by the clinical failure of early stromal depletion approaches. Hedgehog pathway inhibition in preclinical PDAC models revealed that stromal loss could paradoxically accelerate tumor progression, highlighting that some stromal components exert tumor-restraining functions rather than acting as uniformly tumor-promoting elements [104]. Consistent with this, enzymatic stromal depletion with pegvorhyaluronidase alfa (PEGPH20) failed to improve outcomes in metastatic PDAC; in the phase III HALO-301 trial, PEGPH20 plus nab-paclitaxel/gemcitabine did not improve overall survival compared with chemotherapy alone in hyaluronan-high disease [117]. Similarly, in the MORPHEUS platform study, the addition of atezolizumab plus PEGPH20–based regimens did not translate into a clear survival advantage over chemotherapy in advanced PDAC, further underscoring the limitations of stromal depletion strategies [118]. These findings collectively shifted the field away from indiscriminate stromal ablation and toward context-dependent stromal normalization or reprogramming strategies that aim to relieve physical and immunologic barriers while preserving tumor-restraining stromal functions [104]. The immunosuppressive TME remains a major barrier to effective immunotherapy in PDAC and is central to its classification as an immunologically “cold” tumor [47]. Accordingly, current TME-remodeling strategies seek to convert this exclusionary microenvironment into a more inflamed and immune-permissive state by targeting stromal architecture, myeloid-cell function, and tumor-associated metabolic programs [101].

One emerging clinical example is lixumistat, an OxPhos-targeting metabolic inhibitor that is being evaluated in combination with gemcitabine and nab-paclitaxel in a phase 1b study (NCT05497778) in metastatic PDAC; early data identified 400 mg once daily as the recommended phase 2 dose and showed preliminary antitumor activity in this small signal-finding cohort.

Together, these studies reflect a broader therapeutic shift from stromal depletion toward rational TME reprogramming in pancreatic cancer [119].

#### 6.3.1. TGF-β Inhibition Combined with ICIs: Alleviating Fibrosis and Restoring T-Cell Infiltration

TGF-β is a prototypical immunosuppressive cytokine in PDAC that drives fibrosis and T-cell exclusion by activating CAFs and promoting ECM deposition, thereby creating both physical and biochemical barriers to immune infiltration [120]. Preclinical PDAC studies support the concept that TGF-β pathway inhibition, when combined with checkpoint blockade, can suppress neoplastic progression, enhance CD8^+^ T-cell infiltration, and improve therapeutic responsiveness [121]. These data indicate that TGF-β blockade represents an important mechanistic axis for overcoming ICI resistance and provide a strong rationale for its use as a TME-modulating partner that can relax desmoplastic constraints and improve immune accessibility. Nevertheless, clinical translation will require dose optimization and scheduling, as well as the development of predictive biomarkers, such as TGF-β pathway activity and F-TBRS levels, to identify patients most likely to benefit, and to avoid systemic toxicity associated with broad TGF-β inhibition [100,120].

#### 6.3.2. Targeting Myeloid Suppression and Metabolic Checkpoints

MDSCs and TAMs are key cellular components that sustain the immunosuppressive ecosystem of the PDAC TME [122]. The CSF1/CSF1R axis regulates the polarization of macrophages toward an immunosuppressive M2-like phenotype, and its inhibition can reprogram macrophage function and enhance T-cell activity [123]. However, early clinical studies targeting the CSF1/CSF1R axis have yielded limited clinical benefit despite measurable immunologic effects [124], underscoring the need for multi-axis strategies that combine myeloid targeting with additional interventions such as FAK, CXCR4, or TGF-β blockade. The PDAC TME is characterized by profound metabolic suppression driven by hypoxia and nutrient deprivation. Lactate accumulation from aerobic glycolysis contributes directly to extracellular acidification, impairing T-cell activation and promoting M2 polarization of TAMs [125,126]. Importantly, adenosine does not itself create an acidic microenvironment; rather, hypoxia and the resulting acidosis drive the upregulation of CD39 and CD73 on tumor cells, CAFs, and Tregs, which sequentially hydrolyze extracellular ATP to AMP and then to adenosine [85]. Adenosine then acts on A2A receptors on T cells, NK cells, and dendritic cells to broadly suppress effector immune function, independent of its pH effects. The causal sequence is therefore hypoxia/acidosis → CD39/CD73 upregulation → adenosine accumulation → immune suppression, not the reverse [85]. This metabolic immune suppression has led to the conceptualization of metabolic checkpoints, with pathways such as IDO1 (indoleamine 2,3-dioxygenase), the CD73–adenosine axis, and lactate dehydrogenase A (LDH-A) emerging as attractive targets [42,125,127]. These pathways can impose metabolic constraints on effector T-cells and reprogram tryptophan metabolism, thereby driving dysfunctional immune states and weakening antitumor immunity [42,125,127]. Inhibiting these metabolic checkpoints, particularly in combination with ICIs, is being explored as a next-generation immunometabolic strategy to reinvigorate T-cell function and enhance therapeutic responses in PDAC [125,127].

## 7. Future Therapeutic Directions: Controlling the Tumor Ecosystem to Achieve Precision Medicine

PDAC remains one of the most intractable malignancies in modern oncology and is characterized by a uniquely complex ecosystem in which profound ITH is tightly intertwined with a strongly immunosuppressive TME [85,102,126]. This multilayered complexity underpins both the limited clinical efficacy of ICIs and the intrinsic and acquired resistance that defines PDAC [102,126]. In this review, we examined the interplay between ITH and the TME through the integrative lens of “immunity within heterogeneity” and used this framework to interpret the mechanisms of therapeutic resistance and immune unresponsiveness in PDAC [85].

ITH is the central axis of evolutionary adaptability in PDAC. Beyond genetic mutations, epigenetic, transcriptomic, and metabolic plasticity enables tumor subclones to rapidly adapt to therapeutic selective pressures [85,128]. Such clonal diversity generates heterogeneous immune responses and progressively reinforces immune evasion through continuous co-evolution with the TME [85]. The TME comprises CAFs, the ECM, TAMs, MDSCs, Tregs, and other components that form a highly coordinated immunosuppressive ecosystem [102,126]. This ecosystem imposes both physical and immunologic barriers and renders PDAC a prototypical “immune-excluded” tumor [102,126]. Fibrosis, hypoxia, lactate accumulation, and TGF-β signaling, among others, structurally and functionally restrict immune cell infiltration and activation, thereby contributing to the failure of ICI monotherapy [100,102,125,126].

Recent technological advancements, including single-cell multi-omics, spatial transcriptomics, and high-dimensional imaging platforms such as IMC and CODEX, enable quantitative, spatiotemporal dissection of ITH–TME dynamics [80,81,82,84,86,87,88,89,90,91,92,93,94,129,130,131]. In addition, PDOs and immune co-culture systems, together with longitudinal monitoring via ctDNA, provide experimental platforms for tracking tumor evolution and immune interactions in near real time [58,59,60,61,95,96,97,98,99]. These integrated approaches enable a more accurate reconstruction of PDAC biology and lay the groundwork for the development of novel biomarkers that can predict therapeutic responses and resistance [100,105,106,131].

Clinically, therapeutic strategies for PDAC are gradually moving beyond single-agent paradigms toward ITH–TME-guided combination regimens [102,126]. On the clonal axis, targeting of KRAS, PARP, and other DDR-related pathways aims to alleviate tumor-intrinsic mechanisms of immune evasion [17,109,115,132,133]. On the microenvironmental axis, inhibition of TGF-β, FAK, CXCR4, CSF1R, and related stromal or myeloid pathways is being explored to relieve fibrosis and immune exclusion [100,101,120,121,122,123,124,134,135]. Immunometabolic strategies that targeting lactate/adenosine metabolism and pathways such as IDO1 and LDH-A are likewise emerging as next-generation combination partners [42,125,127]. However, the efficacy of these potent agents may depend not only on target engagement but also on overcoming the spatial and biophysical constraints imposed by the PDAC ecosystem. Dense desmoplasia and elevated interstitial fluid pressure can limit intratumoral drug penetration, contributing to suboptimal exposure and systemic toxicity [15]. In this context, therapeutic efforts may benefit more from stromal normalization than indiscriminate stromal depletion, using modulators such as TGF-β or FAK inhibitors to relax extracellular matrix architecture and restore vascular perfusion. Such stromal priming may serve as an enabling step for high-potency agents and could be integrated with sequential or nanoparticle-based delivery strategies to improve tumor-selective exposure while preserving tolerability [14,17,101].

Looking forward, future PDAC therapy is likely to evolve along three major directions:

1. Construction of patient-specific immune atlases through integrated spatial multi-omics: High-resolution, spatially resolved multi-omics profiling will enable patient-level mapping of the structural and functional relationships between ITH and the TME, providing a system-level view of the tumor ecosystem [85,131].
2. Dynamic therapy design based on real-time monitoring of tumor evolution: Longitudinal ctDNA profiling and single-cell sequencing will enable real-time tracking of clonal selection, immune editing, and emergent resistance during treatment, facilitating early detection of therapeutic escape and adaptive modification of therapeutic regimens [58,59,60,61,85].
3. Precision combinatorial immunotherapy: Rational, multi-axis combinations that concurrently target TGF-β, KRAS, CAFs/stroma, and metabolic checkpoints will aim to convert PDAC from an “immune-cold” to a more “immune-hot” tumor, thereby sensitizing it to durable, T-cell-mediated responses [17,100,101,102,125,126,127,136].

To provide a structured overview of these therapeutic opportunities, Table 1 summarizes the top three near-term clinically actionable strategies and the top three speculative future modalities, with attention to mechanism, implementation feasibility, and current obstacles.

Ultimately, the successful management of PDAC will not be achieved by disabling a single dominant target but rather by understanding and re-engineering the co-evolving ecosystem created by tumor subclones and their microenvironment [85,128]. This complexity necessitates the development of the decision-support frameworks illustrated in Figure 3. While the metabolic–epigenetic axis (e.g., histone lactylation) presents a compelling theoretical framework for tumor plasticity, it remains a hypothesis-generating model that requires definitive validation in PDAC-specific clinical cohorts [83,84]. The primary challenge for future precision immuno-oncology lies not only in discovering new targets but in the real-time monitoring of clonal evolution via longitudinal ctDNA and the development of actionable decision-support systems, such as digital twins, that can navigate the immense complexity of the PDAC ecosystem [21,49,94].

Such an ecosystem-centered framework may inaugurate a new paradigm of precision immuno-oncology, not only for PDAC but also for other therapeutically refractory solid tumors [128].

The integrated frameworks outlined in this review—combining KRAS and stromal targeting with chemokine modulation, metabolic checkpoints, ctDNA-guided adaptation, and organoid-based drug testing—are intentionally ambitious and highlight what might ultimately be possible in PDAC [20,28,62,68,69,70,71,79,80,81,82]. However, at present these multi-modal strategies remain clinically underdeveloped: they require rapid, standardized functional platforms, robust biomarker readouts, and coordinated access to multiple agents that are rarely available in routine practice [20,28,58,59,60,61]. Consequently, ctDNA-adapted regimens and organoid-guided treatment switching are best viewed as hypothesis-generating and currently limited to specialized centers and early-phase trials, rather than as broadly deployable standards of care [58,59,60,61,80,81,82]. Explicitly distinguishing these aspirational frameworks from near-term, implementable strategies helps align the conceptual breadth of the review with realistic clinical pathways [20,28,58,59,60,61,79,80,81,82].

In aggregate, the most realistic near-term opportunities in PDAC lie in optimizing chemotherapy-based backbones with one or two carefully chosen targeted or stromal-modulating agents and embedding them into biomarker-enriched trial designs [25,28,33,58,59,60,61,100,101]. By contrast, ecosystem-level strategies that rely on digital twins, spatially targeted CAR-T cells, or metabolic checkpoint interruption should be explicitly framed as longer-term, exploratory approaches that will require substantial methodological and logistical advances before they can be widely implemented [20,28,62,68,69,70,71,79,80,81,82].

## Figures and Tables

**Table 1:** Prioritized therapeutic opportunities in PDAC: Top 3 near-term clinically actionable strategies and Top 3 speculative future modalities.

Therapeutic Axis/Technology	Mechanism of Action	Clinical Implementation & Obstacles
**Top 3 Near-Term Clinically Actionable ^a^**		
1. Direct RAS Inhibition + ICI Priming	KRAS G12D- or pan-RAS–targeted inhibitors suppress intrinsic tumor growth and downregulate immunosuppressive cytokines (for example, GM-CSF, IL-6), potentially rendering “cold” niches more permissive to immune priming.	Currently in phase I–III development; represents a near-term but still early-phase strategy that will require solutions for adaptive bypass signaling and acquired resistance before broad integration with immune checkpoint inhibitors on standard chemo backbones.
2. Biomarker-Enriched PARP Inhibitor Combinations	Exploit homologous recombination repair defects to induce DNA double-strand breaks, activate cGAS–STING signaling, and upregulate PD-L1, thereby sensitizing tumors to ICIs.	Established as a maintenance paradigm in germline BRCA1/2-mutated PDAC but applicable to <5% of unselected patients, so its clinical impact will remain concentrated in tightly molecularly defined niches and requires rigorous upfront genomic screening.
3. Tactically Scheduled TGF-β/FAK Stromal Priming	Transiently lowers extracellular matrix stiffness and interstitial fluid pressure to “relax” physical stromal barriers before administering systemic ICIs or chemotherapy.	Must be delivered as temporally limited priming rather than continuous dosing to minimize systemic tissue toxicity; past experiences with broad stromal depletion and TGF-β blockade have shown mixed efficacy and non-trivial adverse events, demanding careful trial design.
**Top 3 Speculative Future Modalities ^b^**		
1. Patient-Tailored Immuno-Digital Twins	Build predictive in silico “digital twins” from an individual patient’s multi-omics, spatial, and clinical data to simulate the efficacy of candidate regimens before treatment.	Highly speculative; limited by the lack of standardized, multi-center–validated modeling pipelines, high computational complexity, and uncertain regulatory frameworks for using in silico predictions to guide real-time therapeutic decisions.
2. Spatial Meta-Niche–Targeted Dual-Specific CAR-T	Engineer synthetic T cells that co-target a tumor antigen (for example, mesothelin) and a localized immunosuppressive stromal component (for example, FAP-positive inflammatory CAFs).	Carries a high risk of severe on-target, off-tumor toxicity and faces major challenges related to trafficking and survival of CAR-T cells within dense desmoplastic pockets; currently confined to very early-phase or preclinical exploration.
3. Metabolic Checkpoint Interruption (Lactate/Adenosine)	Use small-molecule inhibitors or monoclonal antibodies targeting pathways such as LDHA, CD73, or A2A receptor to disrupt the acidic, adenosine-rich immunometabolic barrier.	Supported by robust preclinical data, but early clinical trials have shown minimal single-agent efficacy; meaningful benefit will likely require integration into broader multi-axis regimens, raising additional complexity and toxicity-management challenges.

^a^ All three near-term strategies are envisioned as modular add-ons to established chemotherapy backbones rather than stand-alone regimens. ^b^ At present, these speculative modalities are best positioned within hypothesis-generating, early-phase trials at specialized centers rather than as broadly deployable options.

## Data Availability

No new data were created or analyzed in this study. Data sharing is not applicable to this article.

## Abbreviations

The following abbreviations are used in this manuscript:

2-HG	2-hydroxyglutarate
AIRR	adaptive immune receptor repertoire
apCAF	antigen-presenting cancer-associated fibroblast
B2M	*β*2-microglobulin
CAF	cancer-associated fibroblast
ctDNA	circulating tumor DNA
CTL	cytotoxic T lymphocyte
DDR	DNA damage repair
dMMR	mismatch repair-deficient
DTP	drug-tolerant persister
TAM	tumor-associated macrophage
ECM	extracellular matrix
FAK	focal adhesion kinase
F-TBRS	fibroblast TGF-β response signatures
iCAF	inflammatory cancer-associated fibroblast
ICI	immune checkpoint inhibitor
IFN	interferon
IMC	imaging mass cytometry
ITH	intratumor heterogeneity
LDH-A	lactate dehydrogenase A
MDSC	myeloid-derived suppressor cell
MSI-H	microsatellite instability–high
myCAF	myofibroblastic cancer-associated fibroblast
OXPHOS	oxidative phosphorylation
PARPi	PARP inhibitors
PBMCs	peripheral blood mononuclear cells
PDAC	pancreatic ductal adenocarcinoma
PDOs	patient-derived organoids
PSC	pancreatic stellate cell
scRNA-seq	single-cell RNA sequencing
scTCR-seq	single-cell T-cell receptor sequencing
TCA	tricarboxylic acid
Teff	T effector
TILs	tumor-infiltrating lymphocytes
TMB	tumor mutational burden
TME	tumor microenvironment
Treg	regulatory T-cell
α-KG	α-ketoglutarate
